# Addendum: Irrational behavior in *C. elegans* arises from asymmetric modulatory effects within single sensory neurons

**DOI:** 10.1038/s41467-019-12288-1

**Published:** 2019-09-23

**Authors:** Shachar Iwanir, Rotem Ruach, Eyal Itskovits, Christian O. Pritz, Eduard Bokman, Alon Zaslaver

**Affiliations:** Department of Genetics, Silberman Institute of Life Science, Edmond J. Safra Campus, The Hebrew University of Jerusalem, Jerusalem, 9190401 Israel

**Keywords:** Decision, Sensory processing, Animal behaviour

## Abstract

We would like to make our readers aware of the publication by Cohen et al., which reports irrational behaviour in C. elegans olfactory preference[1] . These complementary studies establish C. elegans as a model system to explore the neural mechanisms of decision making.

Addendum to: *Nature Communications* 10.1038/s41467-019-11163-3, published online 19 July 2019.

We would like to make our readers aware of the publication by Cohen et al., which reports irrational behaviour in *C. elegans* olfactory preference^1^. These complementary studies establish *C. elegans* as a model system to explore the neural mechanisms of decision making.
