# Supplementary material for: Irrational behavior in C. elegans arises from asymmetric modulatory effects within single sensory neurons
Source: Nat Commun. 2019 Jul 19;10:3202. doi: 10.1038/s41467-019-11163-3 (PMC6642097; doi:10.1038/s41467-019-11163-3)
Supplement: Supplementary file 1 — Supplementary Information [file 41467_2019_11163_MOESM1_ESM.pdf]

Supplementary Information

Irrational behavior in *C. elegans* arises from asymmetric modulatory effects within single sensory neurons

Shachar Iwanir*, Rotem Ruach*, Eyal Itskovits, Christian O. Pritz, Eduard Bokman, Alon Zaslaver

Department of Genetics, Silberman Institute of Life Science, Edmond J. Safra Campus

The Hebrew University of Jerusalem

*These authors contributed equally to this work

Corresponding author: alonzas@mail.huji.ac.il

<u>Table of contents:</u>

- Supplementary figures
- Supplementary reference

Supplementary figure 1

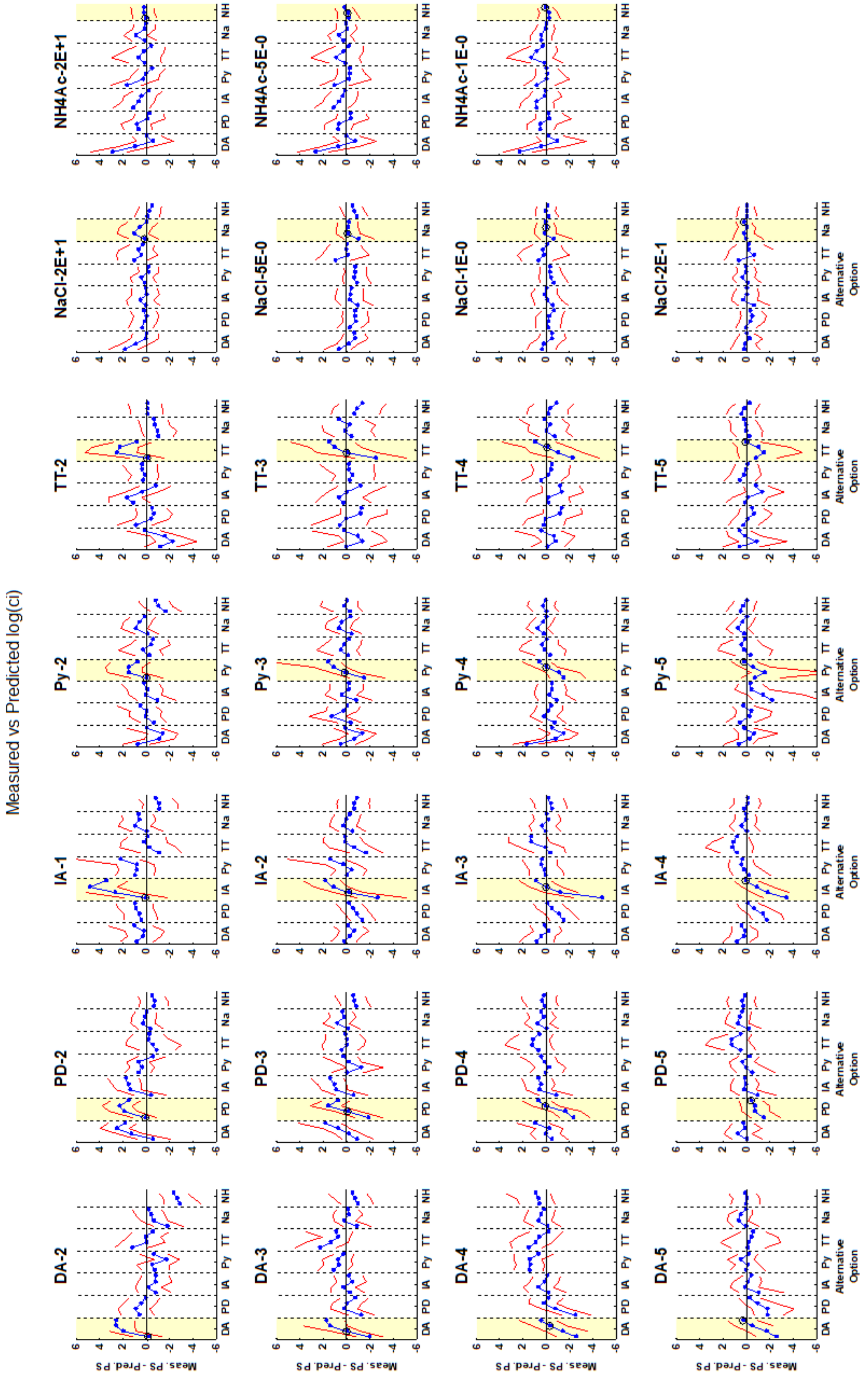

Supplementary figure 1. Differences between measured and predicted 'Pair Scores' reveal stimuli that affect the preference towards other stimuli. Each graph represents the difference between the measured and the predicted 'Pair Scores' of the option given at the title against all other stimuli (alternative options), appearing in the x-axis. The predicted Pair Score is the difference between the basal preference towards the stimulant (BCI, that is, versus no other options) shown in the title and the BCI of the x-axis option. Blue dots and lines represent the difference values, while red lines mark the 95% confidence intervals. A positive value indicates that the stimulus given in the title of each panel is preferred over its alternative option shown in the x-axis, and vice versa. Vertical dashed lines separate the different stimulants as alternative options, in decreasing dose order within each stimulant (-2, -3, -4, -5 for DA, PD, Py and TT; -1, -2, -3, -4 for IA; 20, 5, 1 and 0.2 M for NaCl; 20, 5 and 1 M for NH_4Ac, according to the description in Fig. 1b). Yellow-shaded boxes indicate same-stimulant alternative options. Note the consistent preference towards high over low doses of the same volatile stimulants. A black circle marks the relationship of each option to itself, which essentially approximates zero.

Supplementary figure 2

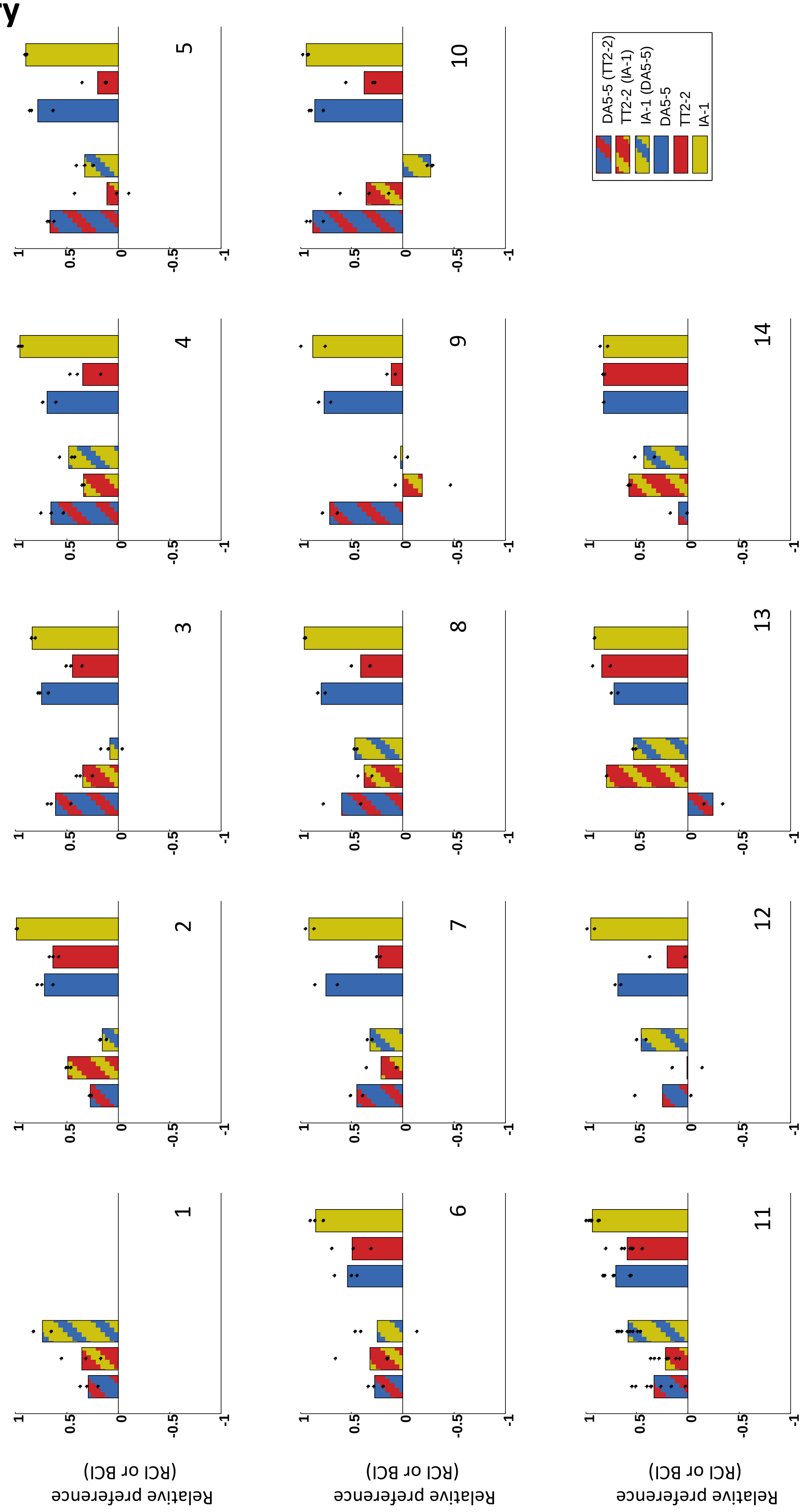

Supplementary figure 2. Intransitivity (violation of WST) in the triple set DA/TT/IA is robustly evident at the single experiment level. Each plot represents a different experimental day (14 in total), and dots indicate the results of individual plate assays from the same experimental day (typically 2 to 3 plates/experiment for each pairwise interaction). Smooth bars represent the Basal Chemotaxis Index (BCI) of each of the three options and dashed bars represent their mutual Relative Chemotaxis Index (RCI). Note that RCIs are mostly positive and are displayed such that each option appears once as the reference option and once as the alternative option. This actually manifests intransitivity (violation of Weak Stochastic Transitivity, WST). In 7/14 (50%) experiments (1,2,4,7,8, 11 and 14) preference ratios in all plates of all groups were positive, supporting WST violation. In three of them (3,5 and 6), only one plate displayed an opposite (negative) behavior, but WST violation was preserved when considering the mean RCIs. In the remaining four experiments, WST was satisfied with at least one interaction displaying an opposite valence than the others. These incidents were typically associated with anomalous performance of at least one relevant option at the BCI level [*e.g.* negative DA5-5(TT2-2) RCI in experiment 13 associated with higher-than-usual TT2-2 BCI; negative TT2-2(IA-1) RCI in experiment 9 associated with a lower-than-usual TT2-2 and higher-than-usual IA-1 BCIs].

Supplementary figure 3

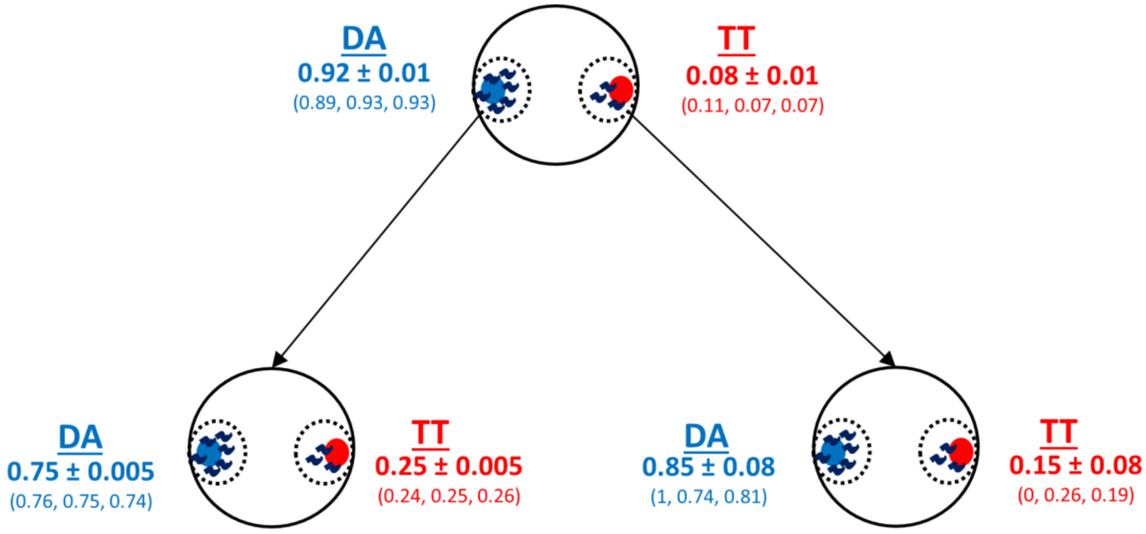

Supplementary figure 3. The intransitivity is not a result of individuals that may have different preference orders (known as the 'voting' or the Condorcet's paradox [1]). According to this paradox, intransitivity at the population level may arise even if individual animals behave rationally. This is because the population is heterogeneous and individuals may have different preference orders which they reliably maintain in repeated tasks. To overrule this possibility, we collected separately worms which chose either TT or DA in their first choice assay. Following 8 hours, we repeated the assay with each group separately. We found that individuals did not have a characteristic preference and that the majority of individuals that chose TT in the first assay, preferred DA in the subsequent choice assay. Shown are the average preference ratios of three independent experimental repeats. In parentheses are the results of the three individual experiments. In the first assay we loaded 300-500 worms, and the subsequent assay typically consisted of 30-100 worms.

Supplementary figure 4

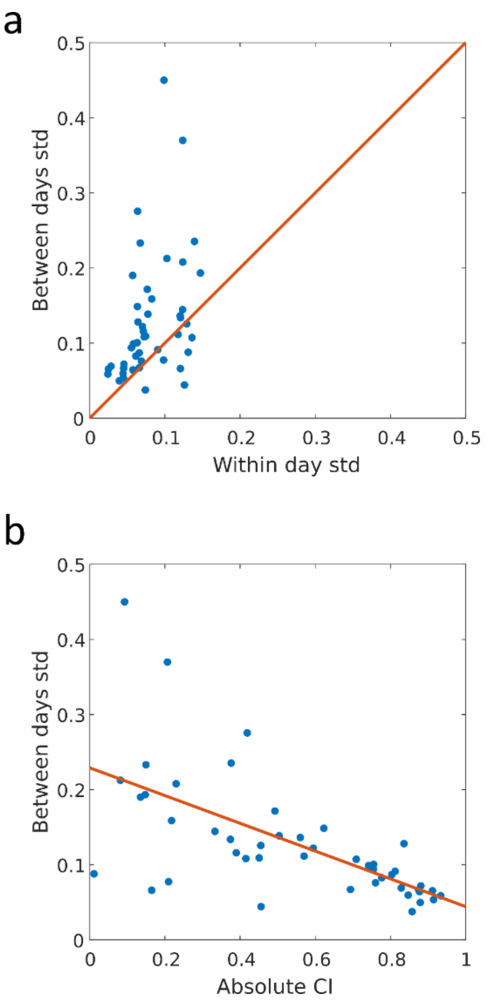

Supplementary Figure 4. Variability analysis of pairwise preference matrix. Of the 406 pairwise combinations consisted of the 28 different stimuli used in the preference matrix (**Fig. 1**), we had 46 representative pairs whose chemotaxis index was measured in at least 5 different days (**Supplementary Table 1**). On each of these days, we included on average 3-4 technical repeats (assay plates) for each of the tested pairs. **(a)** Mean standard deviation of the 46 chemotaxis indexes based on within-days analysis (x-axis) plotted against the standard deviation of the mean chemotaxis indexes measured in each day (between-days STD, y-axis). Red line is the Y=X axis. Variation between days is significantly larger than variation within each day (paired ttest, $p<10^{-45}$). **(b)** Standard deviation between days of the same 46 pairs is plotted as a function of the absolute value of the mean chemotaxis index. Red line is a linear fit of the data (R^2=0.40). We then used this regression line to approximate the standard deviation for pairs in the preference matrix for which we had less than 5 experimental repeats (**Figs. 1-3, Supplementary Tables 1-2**).

Supplementary figure 5

a. DA *vs* TT

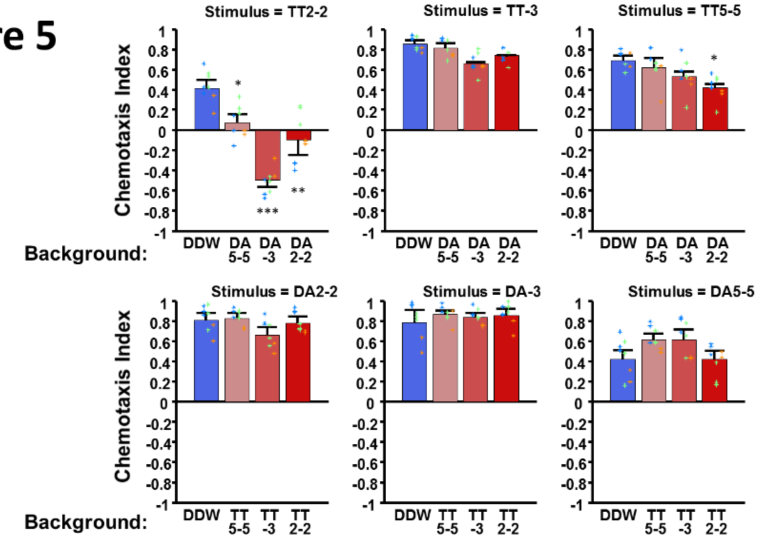

b. TT *vs* IA

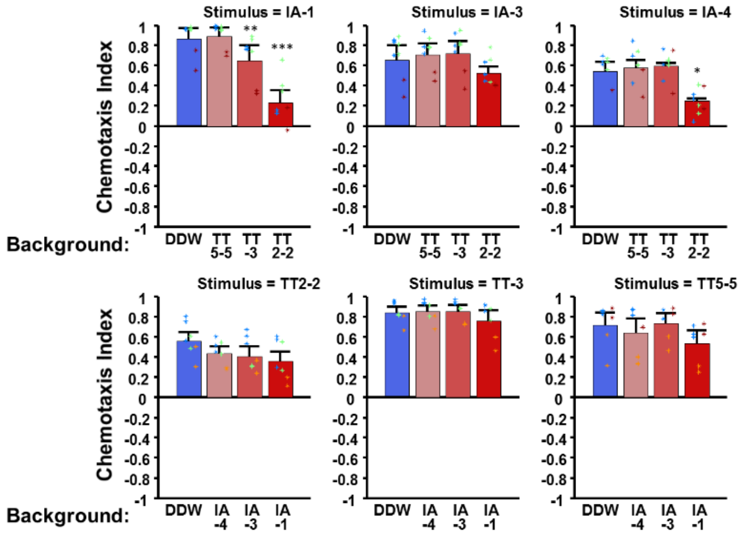

c. IA *vs* DA

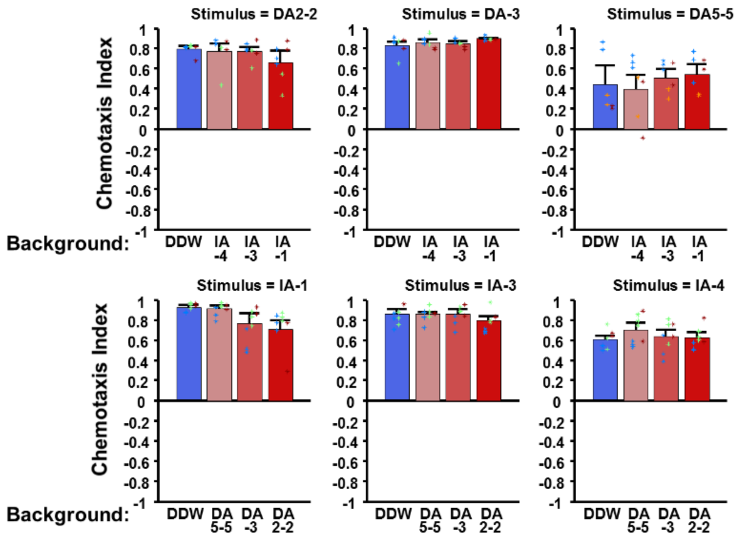

Supplementary Figure 5. Full dose-dependence attraction for TT, DA and IA cross-modulations. Each graph represents the basal chemotaxis index (BCI, Y-axis) towards a target option (provided in the title of each graph) in the background of another cue assayed in three different concentrations (shown in the X-axis). For each pairwise interaction (a,b,c), the bottom row represents the parallel reciprocal interactions of the upper row. **(a) DA/TT cross modulation:** Effect of a DA background on the BCI of various TT doses (top), and of a TT background on the BCI of various DA doses (bottom). **(b) TT/IA cross-modulation:** Effect of TT background on the BCI of various IA doses (top), and of IA background on the BCI of various TT doses (bottom). **(c) IA/DA cross-modulation:** Effect of IA background on the BCI of various DA doses (top), and of DA background on the BCI of various IA doses (bottom). Asterisks denote individual assay plates (2-3 plates per experiments), with experimental days sorted by color and horizontal position. Error bars indicate standard errors of experiments (n=3 for all background/option combinations). */**/*** - significance from the no background control with $p < 0.05/0.01/0.001$, respectively; multivariate linear regression, performed individually for each stimulus with experimental days and background concentrations as parameters. Error bars denote standard error of the mean.

Supplementary figure 6

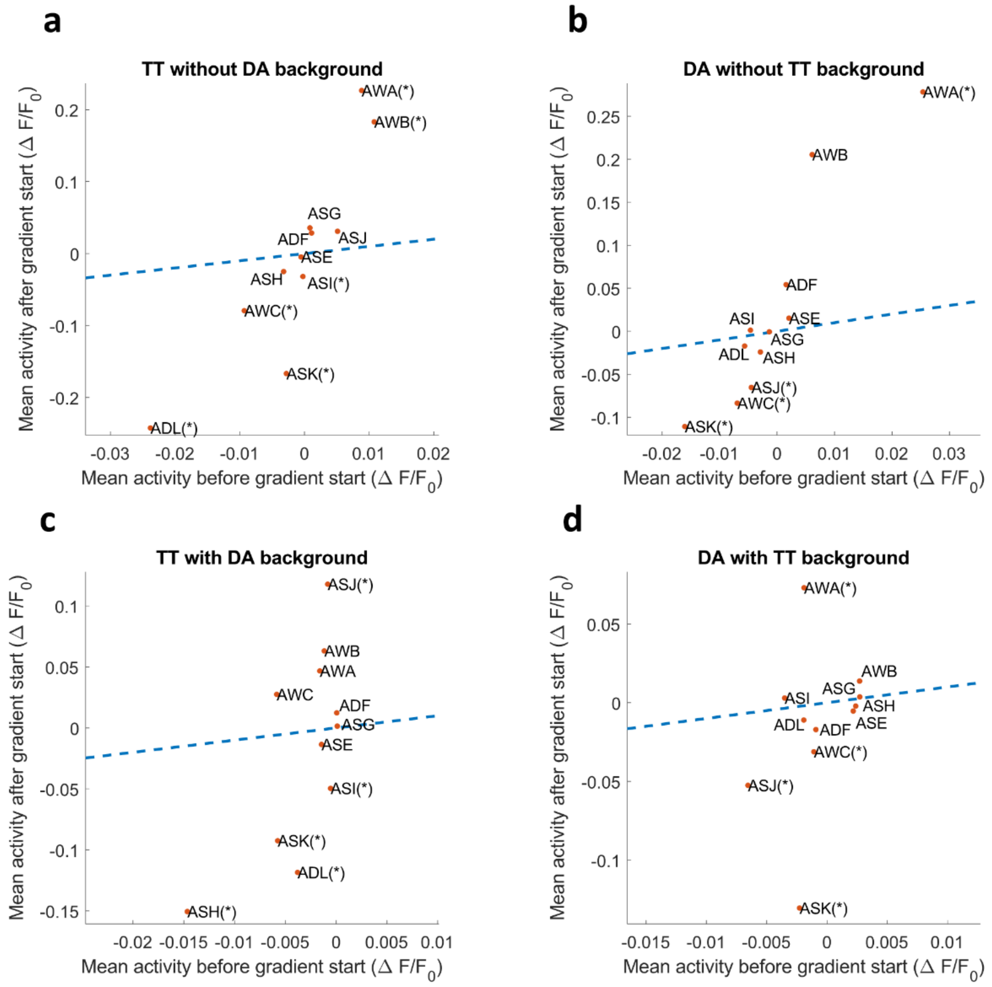

Supplementary Figure 6. Statistical analysis of activated sensory neurons. (a-d) Mean fold change fluorescence in the first 60 seconds after gradient onset (y-axis) is plotted as a function of mean fold change fluorescence in the 50 seconds before gradient start (x-axis) in four experimental conditions: TT/DA gradients without the reciprocal stimulus in the background (a,b) and with the reciprocal stimulus in the background (c,d). Plotted are the mean values of each of the 11 neuron pair types that were imaged in Figure 5 a-d. The 11 neurons in each condition were subjected to Wilcoxon signed-rank test followed by Benjamini-Hochberg FDR correction. (*) marks neuron types whose mean fold-change significantly varied between the two time periods ($p<0.05$). Light sensitive neurons (ASK, ADL, ASH) showed significant reduction in activity throughout the recording due to light adaptation regardless of the gradient. These analyses are based on the *osm-6*::GCaMP reporter strain where all neurons were measured simultaneously. The AWB neurons were also measured using a separate strain with AWB-exclusive expression (as shown in Figure 5i-j).

Supplementary references

1 Gehrlein, W. V. Condorcet's paradox. *Theory and Decision* **15**, 161-197 (1983).
