# Supplementary material for: Irrational behavior in C. elegans arises from asymmetric modulatory effects within single sensory neurons
Source: Nat Commun. 2019 Jul 19;10:3202. doi: 10.1038/s41467-019-11163-3 (PMC6642097; doi:10.1038/s41467-019-11163-3)
Supplement: Supplementary file 3 — Reporting Summary [file 41467_2019_11163_MOESM3_ESM.pdf]

nature research

Corresponding author(s): Alon Zaslaver

Last updated by author(s): Jun 12, 2019

Reporting Summary

Nature Research wishes to improve the reproducibility of the work that we publish. This form provides structure for consistency and transparency in reporting. For further information on Nature Research policies, see Authors & Referees and the Editorial Policy Checklist.

Statistics

For all statistical analyses, confirm that the following items are present in the figure legend, table legend, main text, or Methods section.

n/a	Confirmed	
☐	☒	The exact sample size (*n*) for each experimental group/condition, given as a discrete number and unit of measurement
☐	☒	A statement on whether measurements were taken from distinct samples or whether the same sample was measured repeatedly
☐	☒	The statistical test(s) used AND whether they are one- or two-sided<br>*Only common tests should be described solely by name; describe more complex techniques in the Methods section.*
☐	☒	A description of all covariates tested
☐	☒	A description of any assumptions or corrections, such as tests of normality and adjustment for multiple comparisons
☐	☒	A full description of the statistical parameters including central tendency (e.g. means) or other basic estimates (e.g. regression coefficient) AND variation (e.g. standard deviation) or associated estimates of uncertainty (e.g. confidence intervals)
☐	☒	For null hypothesis testing, the test statistic (e.g. *F*, *t*, *r*) with confidence intervals, effect sizes, degrees of freedom and *P* value noted<br>*Give P values as exact values whenever suitable.*
☒	☐	For Bayesian analysis, information on the choice of priors and Markov chain Monte Carlo settings
☐	☒	For hierarchical and complex designs, identification of the appropriate level for tests and full reporting of outcomes
☐	☒	Estimates of effect sizes (e.g. Cohen's *d*, Pearson's *r*), indicating how they were calculated

Our web collection on statistics for biologists contains articles on many of the points above.

Software and code

Policy information about availability of computer code

Data collection	No software was used.
Data analysis	We used custom Matlab code to analyze the data.

For manuscripts utilizing custom algorithms or software that are central to the research but not yet described in published literature, software must be made available to editors/reviewers. We strongly encourage code deposition in a community repository (e.g. GitHub). See the Nature Research guidelines for submitting code & software for further information.

Data

Policy information about availability of data

All manuscripts must include a data availability statement. This statement should provide the following information, where applicable:

- Accession codes, unique identifiers, or web links for publicly available datasets
- A list of figures that have associated raw data
- A description of any restrictions on data availability

The data are fully available in the supplementary tables. A data availability statement is included in the manuscript.

Field-specific reporting

Please select the one below that is the best fit for your research. If you are not sure, read the appropriate sections before making your selection.

☒ Life sciences ☐ Behavioural & social sciences ☐ Ecological, evolutionary & environmental sciences

For a reference copy of the document with all sections, see nature.com/documents/nr-reporting-summary-flat.pdf

Life sciences study design

All studies must disclose on these points even when the disclosure is negative.

Sample size	For the comprehensive behavioral screen shown in figure 1b, many choice assays were performed only few times resulting in under-sampling. We explicitly state that in the manuscript. To overcome this, we chose 46 representative choice assays and repeated them at least five times. This provided a reliable measure of the experimental variability that we then used to estimate the variation in the under-sampled assays. For the specific condition that we focused on following this initial screen, we performed 14 repeats showing clear reproducibility which further supported the conclusions drawn from these experiments.
Data exclusions	No data were excluded.
Replication	All attempts at replication were successful.
Randomization	Not relevant
Blinding	Not relevant

Reporting for specific materials, systems and methods

We require information from authors about some types of materials, experimental systems and methods used in many studies. Here, indicate whether each material, system or method listed is relevant to your study. If you are not sure if a list item applies to your research, read the appropriate section before selecting a response.

Materials & experimental systems

n/a	Involved in the study
☐	☐ Antibodies
☐	☐ Eukaryotic cell lines
☐	☐ Palaeontology
☐	☒ Animals and other organisms
☐	☐ Human research participants
☐	☐ Clinical data

Methods

n/a	Involved in the study
☐	☐ ChIP-seq
☐	☐ Flow cytometry
☐	☐ MRI-based neuroimaging

Antibodies

Antibodies used	Not relevant
Validation	Not relevant

Eukaryotic cell lines

Policy information about cell lines

Cell line source(s)	Not relevant
Authentication	Not relevant
Mycoplasma contamination	Not relevant
Commonly misidentified lines (See ICLAC register)	Not relevant

Palaeontology

Specimen provenance	Not relevant
Specimen deposition	Not relevant
Dating methods	Not relevant

☐ Tick this box to confirm that the raw and calibrated dates are available in the paper or in Supplementary Information.

Animals and other organisms

Policy information about studies involving animals; ARRIVE guidelines recommended for reporting animal research

Laboratory animals	C. elegans worms. Young adult hermaphrodites.
Wild animals	Not relevant
Field-collected samples	Not relevant
Ethics oversight	Not relevant

Note that full information on the approval of the study protocol must also be provided in the manuscript.

Human research participants

Policy information about studies involving human research participants

Population characteristics	Not relevant
Recruitment	Not relevant
Ethics oversight	Not relevant

Note that full information on the approval of the study protocol must also be provided in the manuscript.

Clinical data

Policy information about clinical studies

All manuscripts should comply with the ICMJE guidelines for publication of clinical research and a completed CONSORT checklist must be included with all submissions.

Clinical trial registration	Not relevant
Study protocol	Not relevant
Data collection	Not relevant
Outcomes	Not relevant

ChIP-seq

Data deposition

☐ Confirm that both raw and final processed data have been deposited in a public database such as GEO.

☐ Confirm that you have deposited or provided access to graph files (e.g. BED files) for the called peaks.

Data access links<br>*May remain private before publication.*	Not relevant
Files in database submission	Not relevant
Genome browser session<br>(e.g. UCSC)	Not relevant

Methodology

Replicates	Not relevant
Sequencing depth	Not relevant
Antibodies	Not relevant
Peak calling parameters	Not relevant
Data quality	Not relevant
Software	Not relevant

Flow Cytometry

Plots

Confirm that:

- [ ] The axis labels state the marker and fluorochrome used (e.g. CD4-FITC).
- [ ] The axis scales are clearly visible. Include numbers along axes only for bottom left plot of group (a 'group' is an analysis of identical markers).
- [ ] All plots are contour plots with outliers or pseudocolor plots.
- [ ] A numerical value for number of cells or percentage (with statistics) is provided.

Methodology

Sample preparation	Not relevant
Instrument	Not relevant
Software	Not relevant
Cell population abundance	Not relevant
Gating strategy	Not relevant

- [ ] Tick this box to confirm that a figure exemplifying the gating strategy is provided in the Supplementary Information.

Magnetic resonance imaging

Experimental design

Design type	Not relevant
Design specifications	Not relevant
Behavioral performance measures	Not relevant

Acquisition

Imaging type(s)	Not relevant
Field strength	Not relevant
Sequence & imaging parameters	Not relevant
Area of acquisition	Not relevant

Diffusion MRI ☐ Used ☐ Not used

Preprocessing

Preprocessing software	Not relevant
Normalization	Not relevant
Normalization template	Not relevant
Noise and artifact removal	Not relevant
Volume censoring	Not relevant

Statistical modeling & inference

Model type and settings	Not relevant
Effect(s) tested	Not relevant

Specify type of analysis: ☐ Whole brain ☐ ROI-based ☐ Both

Statistic type for inference (See Eklund et al. 2016)	Not relevant
Correction	Not relevant

Models & analysis

n/a	Involved in the study
☐	☐ Functional and/or effective connectivity
☐	☐ Graph analysis
☐	☐ Multivariate modeling or predictive analysis

Functional and/or effective connectivity	Not relevant
Graph analysis	Not relevant
Multivariate modeling and predictive analysis	Not relevant
