# Supplementary material for: Irrational behavior in C. elegans arises from asymmetric modulatory effects within single sensory neurons
Source: Nat Commun. 2019 Jul 19;10:3202. doi: 10.1038/s41467-019-11163-3 (PMC6642097; doi:10.1038/s41467-019-11163-3)
Supplement: Supplementary file 4 — Description of Additional Supplementary Files [file 41467_2019_11163_MOESM4_ESM.pdf]

Description of Additional Supplementary Files

File Name: Supplementary Data 1
Description: Results summary of the comprehensive binary preferences experiments.

File Name: Supplementary Data 2
Description: Rationality classification of all examined triplets.
